# Impact of Aerobic Training on Cardiovascular Function, Fitness, and Patient Reported Outcomes During Anthracycline Chemotherapy: A Case Series in Women With Breast Cancer

**DOI:** 10.1177/15347354231210874

**Published:** 2023-11-14

**Authors:** James Murray, Hunter Bennett, Sudarsha Selva-Nayagam, Rohit Joshi, Eva Bezak, Rebecca Perry

**Affiliations:** 1 Allied Health and Human Performance, University of South Australia, Adelaide, SA, Australia; 2 Cancer Centre, Royal Adelaide Hospital, Adelaide, SA, Australia; 3 Cancer Centre, Lyell McEwin Hospital, Adelaide, SA, Australia; 4 Department of Physics, University of Adelaide, Adelaide, SA, Australia

**Keywords:** physical activity, global longitudinal strain, echocardiography

## Abstract

**Background:**

Chemotherapy for breast cancer can increase the risk of cancer therapy related cardiac dysfunction (CTRCD). Exercise has been proposed to prevent CTRCD, however, research to date has indicated high degrees of individual variability following exercise interventions in this population.

**Aim:**

This study aimed to explore the impact of regular, individualized aerobic exercise on CTRCD incidence (defined by global longitudinal strain [GLS]) during and immediately upon the completion of dose-dense anthracycline (DDAC) chemotherapy in 5 women with breast cancer.

**Methods:**

Five women receiving DDAC with stage I-III breast cancer enrolled. Participants underwent resting echocardiography and exercise testing before, during, upon the completion of, and 3 months after the completion of DDAC treatment to measure GLS and aerobic fitness (VO_2_peak). Participants opted-in to an individualized 8-week aerobic exercise intervention (3 sessions per week, 24 sessions total) or standard care for the duration of their DDAC treatment. Data for each participant were presented descriptively.

**Results:**

Four of the 5 participants completed the exercise intervention during DDAC treatment (adherence 79.2%-91.7%). Mild asymptomatic CTRCD occurred in 2 of the 4 exercising participants, of whom both were at an increased risk (one was >65 years of age and diagnosed with hypertension, with the other receiving trastuzumab prior to DDAC treatment). Varied responses in VO_2_peak were observed and did not align with changes in GLS. The only participant not to complete the exercise intervention reported poorer health related quality of life and increased cancer related fatigue at all measurement timepoints.

**Conclusion:**

This study details the individual variability in cardiovascular responses to exercise that can occur during DDAC treatment in women with breast cancer, which can inform exercise professionals and researchers when designing individualized exercise programs for this population.

## Introduction

Breast cancer is the most commonly diagnosed cancer worldwide, with over 2.3 million new cases each year.^1^ While advances in screening and treatment have seen breast cancer mortality rates decline,^2^ the risk of mortality from treatment related side effects, including cancer therapy related cardiac dysfunction (CTRCD), has increased.^3,4^ CTRCD is damage to the heart caused by the toxic properties of certain chemotherapeutic agents, and can present as left ventricular dysfunction, cardiomyopathy, or heart failure.^5^ Anthracyclines (AC) are one of the most common chemotherapeutic agents associated with CTRCD, with an incidence of approximately 10% in patients treated with AC.^6,7^ However, they are also one of the most common and effective breast cancer treatments.^8^ As such, interventions that can reduce the negative impact of AC treatment on cardiovascular function may assist in the prevention of CTRCD.

Cardiac monitoring via echocardiography is recommended as part of standard care in patients undergoing AC treatment, with recent European Society of Cardiology guidelines providing updated criteria for the diagnosis of CTRCD.^9^ CTRCD is now classified broadly as asymptomatic or symptomatic. Asymptomatic CTRCD can be categorized as mild (left ventricular ejection fraction [LVEF] ≥50% and relative decline in global longitudinal strain [GLS] by ≥15% from baseline), moderate (reduction in LVEF by ≥10 percentage points to an LVEF of 40% to 49%, or reduction in LVEF of <10 percentage points to an LVEF of 40% to 49% with a relative decline in GLS by ≥15% from baseline), or severe (reduction in LVEF to <40%).^9^ This is the first guideline to include GLS in the diagnosis of CTRCD, with GLS recognized to detect early changes in myocardial function prior to changes in LVEF, and to be predictive of future CTRCD in patients treated with cardiotoxic anti-cancer therapies.^5,10^ The inclusion of GLS in CTRCD diagnosis will allow the implementation of preventative therapies before a significant decline in LVEF occurs, which in some cases can be irreversible, and interrupt anti-cancer therapy.^9,11^

Exercise is a well-recognized intervention that can improve cardiovascular health and function and reduce the risk of modifiable risk factors associated with the development of CTRCD including hypertension, diabetes, and dyslipidaemia.^12,13^ Evidence also supports the use of exercise to improve cardiorespiratory fitness, cancer related fatigue, and quality of life in individuals with breast cancer, and has been shown to be safe and feasible at all stages across the cancer continuum.^14-17^ As such, there is growing interest in the ability of exercise to attenuate declines in GLS and LVEF and prevent CTRCD in patients undergoing cardiotoxic chemotherapy for the treatment of breast cancer.^18,19^ As GLS is a highly sensitive measure that can detect early changes in myocardial function, and is recognized to precede reductions in LVEF,^5,10^ frequent monitoring of GLS *during* AC chemotherapy may provide the greatest insight into the ability of exercise to prevent asymptomatic CTRCD. A systematic review performed by the current authors found the ability of exercise to prevent CTRCD was unclear due to the small number of studies and large diversity in methodology and interventions between studies in this field.^20^ Included studies in this review were also limited by a lack of reporting of individual data and lack of reporting of CTRCD incidence,^20^ which is a significant limitation given the individual nature of CTRCD.^13^ Further, included studies had either a short, or no follow-up period. A recent randomized trial investigating the impact of exercise on CTRCD prevention in women with breast cancer undergoing AC treatment found that exercise improved cardiac reserve and attenuated declines in aerobic fitness, however, no differences in GLS, LVEF, and CTRCD incidence between a control group were observed 1 month after the completion of treatment.^21^ Conversely, in women with hypertension and diabetes not undergoing cardiotoxic chemotherapy, an 8-week aerobic exercise intervention may improve GLS.^22^ The difference in outcomes between these 2 studies could suggest that cardiotoxic treatment outweighs the positive effect of exercise on GLS, or GLS may not detect improvements in cardiac function following exercise during AC-based chemotherapy.^21^ However, as research in this area is still emerging,^19^ the impact of aerobic exercise on GLS and asymptomatic CTRCD during, and immediately upon the completion of AC chemotherapy remains unknown. As individual responses to similar doses of chemotherapy and exercise vary, and changes in GLS occur early in the course of CTRCD development, a detailed understanding of the short-term, individual responses to exercise during AC treatment is a logical step to further understand the role of exercise in this setting.

Therefore, the following series of case reports aims to present detailed information on how regular, individualized aerobic exercise impacts GLS, asymptomatic CTRCD incidence, aerobic fitness, and patient reported outcomes during and immediately upon the completion of dose-dense AC chemotherapy in women with breast cancer.

## Methods

### Participants and Experimental Design

This paper presents case studies of 5 women who underwent dose-dense (DD) AC chemotherapy between January 2022 and March 2023. Participants were eligible for inclusion if they were: female, scheduled to begin adjuvant or neoadjuvant DDAC chemotherapy for the treatment of stage I-III breast cancer, and had no history of structural heart disease or arrythmias. Participants were recruited from 3 hospital sites in South Australia (Royal Adelaide Hospital, Lyell McEwin Hospital, Calvary North Adelaide Hospital), and referred into the study by their treating oncologist. This study was granted ethics approval by the Central Adelaide Local Health Network Human Research Ethics Committee (protocol number 13976) and the University of South Australia Human Research Ethics Committee (protocol number 203909), and prospectively registered with the Australian New Zealand Clinical Trials Registry (ANZCTR) (ACTRN12621001030864). All experimental procedures were explained to participants, with signed, informed consent obtained prior to partaking in any research activities. All procedures conformed to the standards set by the Declaration of Helsinki.^23^ Upon enrollment into the study, participants had a choice to join 1 of 2 groups: exercise or standard care. Outcomes measures were collected at 4 timepoints throughout the study; baseline (the week before commencing DDAC chemotherapy), 4 weeks (after 2 cycles of DDAC chemotherapy), 8 weeks (completion of DDAC chemotherapy), and 5 months (3 months after the completion of DDAC chemotherapy).

### Measurements

#### Participant history and chemotherapy details

The name, prescribed dose, and schedule of all chemotherapy treatments, including DDAC, for each participant was provided by the treating oncologist. Surgery status and outcomes (if performed), medical history, including comorbidities, current non-cancer medications, and smoking history was collected from all participants during the baseline visit of the study. Self-reported physical activity was collected using the International Physical Activity Questionnaire—Short Form.^24^

#### Body composition and point of care testing

Participant height (m) and mass (kg) were measured using a wall stadiometer and calibrated SECA 703 electronic scales (Hamburg, Germany). Body mass index (BMI) was calculated (kg/m^2^). Resting blood pressure was measured using an OMROM HEM 7320 blood pressure monitor (Kyoto, Japan). Non-fasted blood glucose was measured using an Accu-Check Performa Meter (Indianapolis, USA).

#### Patient reported outcomes

The European Organisation for Research and Treatment of Cancer (EORTC) quality of life questionnaire (QLQ)-C30^25^ and QLQ-FA12^26^ were administered to subjectively measure health-related quality of life and cancer related fatigue. The QLQ-C30 consisted of 30 questions across 3 scales, with each scale consisting of multiple domains (global health and quality of life scales, functional scales [physical, role, emotional, cognitive, social], and symptom scales [fatigue, nausea and vomiting, pain, dyspnea, insomnia, appetite loss, constipation, diarrhea, financial difficulties]). The QLQ-FA12 consisted of 12 questions across 5 domains (physical fatigue, emotional fatigue, cognitive fatigue, interference with daily life and social sequelae). All questions were rated from 1 to 4 (1 = “not at all”, 2 = “a little”, 3 = “quite a bit”, 4 = “very much”). Raw scores from each individual domain of the QLQ-C30 and QLQ-FA12 questionnaire were transformed onto a linear 0 to 100 scale for interpretation. For each domain of the QLQ-C30, a score closer to “100” indicated better quality of life and functional outcomes, but worse symptoms,^27^ with a score closer to “100” for each domain of the QLQ-FA12 indicating higher levels of fatigue/interference.^26^ Further information regarding scoring of questionnaires can be found in the relevant scoring manuals.^26,27^ An academic user agreement was in place with the EORTC for the use of each questionnaire.

#### Resting echocardiography

A comprehensive resting echocardiography assessment was performed using commercially available equipment (GE E95, General Electric, Horten, Norway).^28^ LV end-diastolic and end-systolic volumes and LV ejection fraction were measured according to standard guidelines.^29^ LV focused views from an apical 4 chamber, 2 chamber and long axis view of at least 3 beats in duration were acquired at a frame rate of 60 to 90 frames per second for assessment of GLS. Furthermore, a 3D dataset incorporating all LV walls was acquired from an apical 4 chamber position with volume rate adjusted to at least 20 volumes per second. GLS and 3D volumes were calculated using dedicated analysis software on EchoPac (LV Automated Function Imaging analysis and Auto 4D LV analysis, V.204, General Electric, Horten, Norway). For GLS calculation, a region of interest was applied to the endocardial and epicardial borders of the LV with the tracking manually adjusted to cover the entire myocardium over the cardiac cycle. GLS was defined as the change in length in the longitudinal plane of the LV myocardium from end diastole to end systole over the 3 apical views. 3D volumes and LVEF were calculated from the 3D dataset with a region of interest applied to the endocardial border of the LV. This border was tracked by the software over the cardiac cycle and manual adjustment made where required. Participants were deemed inadequate for GLS assessment where 2 or more LV regions were unable to be visualized and tracked. Asymptomatic CTRCD incidence was defined as per current guidelines.^9^

#### Cardiorespiratory fitness

A symptom limited peak exercise test was performed on a Lode Corival (v5.4.0, Groningen, Netherlands) electronically braked stationary recumbent cycle ergometer to measure peak oxygen uptake (VO_2_peak). The test followed an incremental ramp protocol, beginning at 25 Watts (W), and increasing by 15 W each minute until volitional exhaustion. A 1-minute warm up and 3-minute cool-down was completed at 0 W. Respiratory gas (O_2_ and CO_2_) was measured continuously via a facemask connected to a Parvomedics Trueone 2400 Metabolic Measurement System (Utah, United States). VO_2_peak was defined as the highest VO_2_ recorded before volitional exhaustion. Heart rate (HR) was measured using a Polar H10 (Kempele, Finland) wireless chest strap, and recorded in the last 10 seconds of each minute. Rating of perceived exertion (RPE) was measured using a Borg 6 to 20 RPE scale,^30^ and recorded in the last 10 seconds of each minute.

### Interventions

#### Exercise intervention

Participants who selected the exercise arm performed 3 aerobic exercise sessions per week, for 8 consecutive weeks. Each session involved 30 minutes of continuous cycling on a Lode Corival (v5.4.0) electronically braked stationary recumbent cycle ergometer. Exercise intensity was prescribed using a percentage of participant’s peak HR obtained during baseline VO_2_peak testing. The relative intensity of each session was moderate (between 60% and70% HRpeak), with exact intensity adjusted on a fortnightly basis to align with DDAC treatment schedules. Lower relative exercise intensities (60%-65% HRpeak) were prescribed in the first 3 sessions after DDAC treatment, with higher relative exercise intensities prescribed in the 3 sessions performed during the non-treatment week (70% HRpeak). This periodization allowed for reduced relative intensities when fatigue typically peaks post chemotherapy infusion.^31,32^ Relative exercise intensity was re-adjusted after the 4-week testing timepoint based on the new peak HR achieved. All exercise sessions were delivered one-on-one and supervised by an accredited exercise physiologist. HR and RPE were recorded every 5 minutes to monitor exercise intensity and for safety purposes. Resting blood pressure or blood glucose was measured before and after each exercise session in participants with HTN or T2DM, respectively. All participants in the exercise arm were instructed to continue with their daily activities as per normal and were not precluded from performing exercise independently of the study.

#### Standard care

Participants who selected the standard care arm did not perform the exercise intervention described above. Participants in this arm were instructed to continue with their daily activities as normal and were not precluded from performing physical activity and/or exercise independently of the study during their DDAC treatment. Self-reported physical activity was recorded at each measurement timepoint to account for any independent physical activity and/or exercise (see section 2.2.1).

### Statistical Analysis

As data is presented case by case, no grouped analysis was performed. All variables are tabulated and presented as raw values. Relative change from baseline was calculated for echocardiography and cardiorespiratory fitness variables and presented as a percentage (%). Adherence to exercise training frequency was determined by calculating the ratio of attended exercise sessions to the total planned exercise sessions, expressed as a percentage. Adherence to prescribed exercise intensity was calculated by summing the average % of peak HR achieved across each individual exercise session divided by the total number of exercise sessions attended.

## Results

### Participant Demographics and Exercise Adherence

Eighteen women with breast cancer scheduled to begin DDAC treatment were referred, with 6 enrolling, and 5 completing the study. One participant withdrew after enrollment due to an inability to travel to the research facility. The demographic characteristics of each participant at baseline are presented in Table 1.

**Table 1.** Baseline Characteristics of Participants.

	Participant 1	Participant 2	Participant 3	Participant 4	Participant 5
Age, y	68	38	55	45	52
Height, cm	155.4	162.3	163.0	158.0	162.5
Weight, kg	51.7	56.9	75.8	76.35	82.3
BMI, kg·m^2^	21.5	21.7	28.5	30.6	31.0
BSA, m^2^	1.49	1.60	1.82	1.78	1.88
Ethnicity	Asian	Caucasian	Caucasian	Caucasian	Caucasian
Comorbidities	Hypertension	nil	nil	nil	nil
Medications	Telmisartan, 40 mg	nil	nil	nil	nil
Smoking history	nil	nil	nil	nil	nil
Systolic BP, mmHg	126	109	124	130	144
Diastolic BP, mmHg	83	85	74	76	88
Breast cancer					
Stage	IIIa	II	II	Ib	IIb
Tumor immunohistology	n/a	n/a	TNBC	ER/PR/HER2 positive	ER/PR positive, HER2 negative
Chemotherapy setting	Adjuvant	Adjuvant	Neoadjuvant	Neoadjuvant	Neoadjuvant
Surgery type	Mastectomy – Left breast + axillary clearance	Mastectomy – Right breast + axillary clearance	n/a	n/a	n/a
Chemotherapy protocol	Doxorubicin, 60 mg/m^2^, 4 cycles; Cyclophosphamide, 600 mg/m^2^, 4 cycles; Paclitaxel, 80 mg/m^2^, 10 cycles + 60 mg/m^2^, 2 cycles (after DDAC).	Doxorubicin, 60 mg/m^2^, 4 cycles; Cyclophosphamide, 600 mg/m^2^, 4 cycles; Paclitaxel, 80 mg/m^2^, 5 cycles + 70 mg/m^2^, 7 cycles (after DDAC).	Doxorubicin, 60 mg/m^2^, 3 cycles; Cyclophosphamide, 600 mg/m^2^, 3 cycles; Paclitaxel 80 mg/m^2^, 12 cycles (prior to DDAC); Carboplatin, 225 mg/m^2^ (prior to DDAC).	Doxorubicin, 60 mg/m^2^, 4 cycles; Cyclophosphamide, 600 mg/m^2^, 4 cycles; Paclitaxel, 80 mg/m^2^, 12 cycles (prior to DDAC).	Doxorubicin, 60 mg/m^2^, 4 cycles; Cyclophosphamide, 600 mg/m^2^, 4 cycles; Paclitaxel, 80 mg/m^2^, 12 cycles (prior to DDAC).
Targeted therapy	nil	nil	nil	Trastuzumab, 8 mg/kg, 1 cycle + 6/mg/kg, 3 cycles, intravenous (infusion every 3 wk alongside paclitaxel, prior to DDAC).	nil
Exercise group	Yes	Yes	No	Yes	Yes

Abbreviations: y. years; cm, centimeters; kg, kilograms; BMI, body mass index; kg.m^2^, kilograms per meter squared; BSA, body surface area; m^2^, meters squared; mg, milligrams; BP, blood pressure; mmHg, millimeters of mercury; n/a, not applicable; TNBC, triple negative breast cancer; ER, estrogen receptor; PR, progesterone reception; HER2, human epidermal growth factor receptor 2; DDAC, dose dense anthracyclines.

Four participants were middle-aged (range 38-55 years) with 1 older adult (68 years). Based on BMI, 2 participants were classified as healthy, 1 as overweight, and 2 as obese class I. Participant 1 was medicated for hypertension, with all other participants having no comorbidities. All participants underwent DDAC treatment, with 2 in the adjuvant setting and 3 in the neoadjuvant setting. The 2 participants in the adjuvant setting underwent taxane-based treatment following DDAC, with the 3 participants in the neoadjuvant setting undergoing taxane-based treatment prior to DDAC. Participant 4 was the only participant to receive trastuzumab. Participant 3 made the decision to cease DDAC after treatment cycle 3, due to chemotherapy-related toxicity (non-CTRCD related), with all other participants completing 4 cycles (Table 1).

Participants 1, 2, 4, and 5 completed the 8-week exercise intervention during DDAC treatment, with participant 3 opting to complete the standard care arm. Adherence to training frequency was good, with a minimum of 19 and maximum of 22 sessions attended out of a possible 24 (participant 1—91.7% adherence, participant 2—79.2% adherence, participant 4—79.2% adherence, participant 5—87.5% adherence). Adherence to prescribed exercise intensity was also good, with participants averaging between 65% and 71% of HRpeak across the exercise intervention (participant 1—65% HRpeak, participant 2—71% HRpeak, participant 4—68% HRpeak, participant 5—70% HRpeak). No serious adverse events (defined as events resulting in death, persistent or significant disability/incapacity, life-threatening situations, or requiring inpatient hospitalization or prolongation of existing hospitalization) or non-serious adverse events (including lightheadedness, dizziness, drop in blood pressure, chest pain, musculoskeletal pain, or nausea), were reported during or following any exercise session.

### Resting Echocardiography

**Figure 1.**
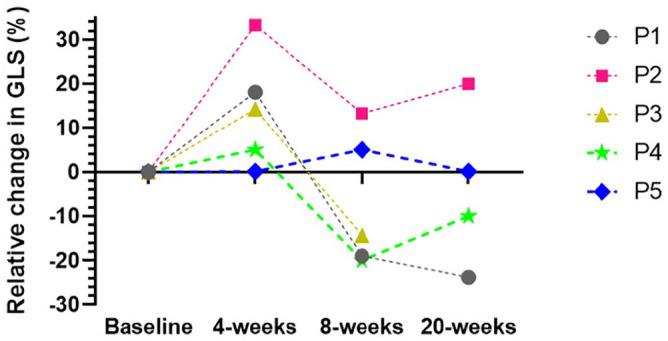
Relative change in GLS from baseline at each time point for each participant. At 4-weeks, GLS improved or was maintained from baseline in all participants, however, at 8-weeks, GLS reduced below the 4-week measurement in all but one participant. Abbreviations: P, participant; GLS, global longitudinal strain.

Resting echocardiography outcomes are presented in Table 2. Mild asymptomatic CTRCD (relative reduction in GLS by ≥15% from baseline and LVEF ≥ 50%) occurred in 2 participants upon the completion of DDAC treatment (participant 1—19% relative reduction in GLS from baseline, participant 4—20% relative reduction in GLS from baseline). In participant 1, GLS reduced further 3 months after the completion of DDAC, with LVEF remaining the same, with participant 4 experiencing an improvement in GLS 3 months after the completion of DDAC, with LVEF remaining the same. Although not meeting the criteria for mild asymptomatic CTRCD, participant 3 (non-exercising participant) experienced a 14.3% relative reduction in GLS from baseline upon completion of DDAC, with a maintenance in LVEF. The remaining 2 participants experienced an improvement in GLS upon the completion of DDAC (participant 2—13% relative increase from baseline, participant 5—5% relative increase from baseline), and did not meet any criteria for asymptomatic CTRCD. Relative change in GLS from baseline at each time point for each participant is presented in Figure 1. No participants experienced a drop in LVEF by ≥10 percentage points or had an LVEF of 40% to 49% at any timepoint. Upon the completion of DDAC treatment, resting HR increased substantially from baseline in participant 1 (23 bpm), participant 2 (12 bpm), participant 3 (23 bpm), and participant 4 (26 bpm).

**Table 2.** Resting Echocardiography and Cardiorespiratory Fitness Outcomes at Baseline, 4-Weeks, 8-Weeks, and 20-Weeks for Each Participant.

	Resting echocardiography								Cardiorespiratory fitness						
	GLS, %	LVEF, %	RVFWS, %	EDV, mL	ESV, mL	HR, bpm	SV, mL	CO, L/min	VO_2_Peak, mL/kg/min	VO_2_Peak, L/min	Peak HR, bpm (% APMHR)	RER	Peak RPE, /20	SBP, mmHg	DBP, mmHg
Participant 1															
Baseline	−21.0	63	−27.0	77	28	64	52.86	3.38	16.3	0.84	137 (85)	1.13	15	126	83
4-weeks	−24.8	61	−24.5	73	28	66	79.97	5.28	16.8	0.86	152 (95)	1.12	13	129	84
8-weeks	−17.0	56	−21.0	71	31	87	54.69	4.76	14.8	0.74	149 (93)	1.18	14	123	81
20-weeks	−16.0	56	−25.0	72	32	73	68.58	5.01	13.4	0.74	134 (84)	1.09	14	98	78
Participant 2															
Baseline	−15.0	60	n/a	74	29	69	51.18	3.53	35.5	2.02	187 (103)	1.17	17	109	85
4-weeks	−20.0	60	−25.0	83	34	68	67.20	3.29	35.1	1.94	184 (101)	1.31	19	103	69
8-weeks	−17.0	59	−18.0	63	26	81	61.55	4.99	31.3	1.78	188 (104)	1.28	19	108	69
20-weeks	−18.0	55	−23.0	75	34	89	53.28	4.74	31.3	1.87	173 (95)	1.21	19	101	70
Participant 3*															
Baseline	−14.0	60	n/a	81	32	60	75.36	4.52	13.1	1.00	113 (67)	1.14	19	124	74
4-weeks	−16.0	58	−20.0	76	32	90	73.05	6.57	13.6	1.04	125 (74)	1.10	15	127	88
8-weeks	−12.0	60	−18.0	79	32	83	68.45	5.68	13.6	1.05	124 (73)	1.05	20	118	82
20-weeks	n/a	n/a	n/a	n/a	n/a	n/a	n/a	n/a	n/a	n/a	n/a	n/a	n/a	n/a	n/a
Participant 4															
Baseline	−20.0	67	−20.0	116	38	60	87.92	5.28	24.8	1.90	150 (85)	1.16	17	130	76
4-weeks	−21.0	60	−19.0	118	47	60	85.41	5.12	24.0	1.81	148 (84)	1.22	15	116	75
8-weeks	−16.0	62	−15.0	98	37	86	66.12	5.69	24.6	1.86	149 (84)	1.10	16	114	80
20-weeks	−18.0	62	−27.0	103	39	59	75.00	4.44	26.0	1.94	160 (91)	1.21	15	129	91
Participant 5															
Baseline	−20.0	56	−26.0	104	45	56	116.26	6.51	21.6	1.77	127 (74)	1.08	19	144	88
4-weeks	−20.0	61	−29.0	124	48	59	101.06	5.96	22.1	1.79	132 (77)	1.05	19	130	85
8-weeks	−21.0	64	−30.0	114	42	56	102.58	5.74	24.2	1.94	141 (82)	1.00	17	118	80
20-weeks	−20.0	59	−21.0	127	52	50	118.00	5.92	22.6	1.09	145 (85)	1.12	17	130	84

Abbreviations: GLS, global longitudinal strain; LVEF, left ventricular ejection fraction; RVFWS, right ventricular free wall strain; EDV, end diastolic volume; ESV, end systolic volume; HR, heart rate; SV, stroke volume; CO, cardiac output; mL, milliliters; bpm, beats per minute; L/min, liters per minute; VO_2_Peak, peak oxygen consumption; %APMHR, percentage of age predicted maximum heart rate; RER, respiratory exchange ratio; RPE, ratings of perceived exertion; SBP, systolic blood pressure; DBP, diastolic blood pressure; mL/kg/min, milliliters per kilogram per minute; mmHg, millimeters of mercury.

* Participant 3 opted into the standard care arm and did not perform the exercise intervention.

### Cardiorespiratory Fitness

Symptom limited exercise testing outcomes are presented in Table 2. Participant 1 and 2 experienced a reduction in VO_2_peak from baseline to 8-weeks (participant 1—9.2% relative reduction from baseline, participant 2—11.8% relative reduction from baseline), which remained reduced 3 months following the completion of DDAC treatment. A maintenance in VO_2_peak was observed in participants 3 and 4, with a 12.1% relative improvement in VO_2_peak observed in participant 5 upon the completion of DDAC treatment. Participant 5’s VO_2_peak remained above baseline levels 3 months after the completion of DDAC treatment, despite experiencing a relative reduction in VO_2_peak of 6.6% during that period, with participant 4 experiencing a 5.7% relative increase in VO_2_peak in the 3 months following DDAC treatment. A higher peak HR was achieved in 4 of the 5 participants at 8-weeks, with peak RPE increasing in participant 2 and 3, and reducing in participant 1, 4, and 5 upon the completion of DDAC treatment. A respiratory exchange ratio of 1.00 or greater was achieved by all participants in all exercise tests. Systolic blood pressure was lower in all participants at 8-weeks compared to baseline, with participants 4 and 5 experiencing the greatest absolute reductions of −16 and −26 mmHg, respectively. These reductions in systolic blood pressure were maintained for participants 1 and 2 three months post DDAC treatment, with participant 4 and 5 experiencing in increase in systolic blood pressure back to baseline and 4-week levels, respectively.

### Self-Reported Physical Activity

Self-reported physical activity for each participant at each timepoint is presented in Table 3. At baseline, only participants 4 and 5 met the currently physical activity guidelines of >150 minutes of moderate to vigorous exercise or physical activity per week (including walking). No participant performed vigorous exercise or physical activity during DDAC treatment, with participants 2 and 5 performing vigorous exercise or physical activity after the completion of DDAC treatment. Moderate exercise and physical activity increased from baseline in participant 1 and 2 and reduced from baseline in participant 4 and 5. All moderate exercise and physical activity performed during DDAC treatment in participants 1, 2, 4, and 5 was attributed to the exercise intervention completed in this study (participant 2 performed 4 exercise sessions in the final week of the study, totaling 120 minutes). Participants 1 and 2 reported a reduction in moderate exercise and physical activity in the 3 months following DDAC treatment, with participants 4 and 5 reporting an increase. Participants 2, 3, 4, and 5 reported a reduction in total walking minutes during treatment, which was more pronounced at 4-weeks, with participants 2, 3, 4, and 5 also reporting an increase in weekly sedentary time during DDAC treatment, despite participants 2, 4, and 5 participating in the exercise intervention. A small increase in weekly walking was reported by participant 1 during treatment, with a maintenance in self-reported sedentary behavior.

**Table 3.** Self-reported Physical Activity and Cancer Related Fatigue (QLQ-FA12) Data at Baseline, 4-Weeks, 8-Weeks, and 20-Weeks for Each Participant.

	Self-reported physical activity				QLQ-FA12 questionnaire				
	Vigorous, min/week	Moderate, min/week	Walking, min/week	Sedentary time, min/day	Physical fatigue, /100	Emotional fatigue, /100	Cognitive fatigue, /100	Daily life, /100	Social sequelae, /100
Participant 1									
Baseline	0	15	0	120	0	0	0	0	0
4-weeks	0	90	10	300	0		0	0	0
8-weeks	0	90	15	120	26.67	0	0	0	0
20-weeks	0	30	0	120	0	0	0	0	0
Participant 2									
Baseline	0	0	120	210	20.00	22.22	33.33	33.33	0
4-weeks	0	90	15	300	26.67	22.22	33.33	33.33	0
8-weeks	0	120	75	480	46.67	11.11	16.67	33.33	0
20-weeks	40	0	45	300	40.00	22.22	16.67	33.33	0
Participant 3*									
Baseline	0	0	0	600	66.67	77.78	83.33	100	66.67
4-weeks	0	0	0	840	80.00	77.78	66.67	100	66.67
8-weeks	0	20	0	720	46.67	66.67	33.33	66.67	0
20-weeks	n/a	n/a	n/a	n/a	n/a	n/a	n/a	n/a	n/a
Participant 4									
Baseline	0	140	60	180	26.67	0	16.67	0	0
4-weeks	0	90	40	300	20.00	11.11	16.67	0	0
8-weeks	0	90	45	600	33.33	22.22	16.67	0	0
20-weeks	0	240	160	240	20.00	0	16.67	0	0
Participant 5									
Baseline	80	120	420	180	6.67	0	0	33.33	0
4-weeks	0	90	225	240	6.67	11.11	0	0	0
8-weeks	0	90	300	360	13.33	0	0	33.33	0
20-weeks	40	120	420	300	0	0	0	0	0

Abbreviations: QLQ-FA12, quality of life questionnaire—fatigue 12; min/week, minutes per week; min/day. minutes per day; /100, score out of 100; n/a, participant did not attend.

* Participant 3 opted into the standard care arm and did not perform the exercise intervention.

### Patient Reported Outcomes

Cancer related fatigue data for each participant are reported in Table 3. Participant 3 reported high levels of fatigue and interference of treatment with daily life at all time points, across all domains. Participant 2 experienced slight physical fatigue that increased from baseline over the course of treatment, while fatigue and interference reported in all other domains remained the same or reduced both during and 3-months post treatment. Participant 4 experienced consistent, slight physical fatigue during and 3-months post treatment, with emotional and cognitive fatigue reported. Minimal fatigue and interference were reported by participants 1 and 5 across all domains throughout DDAC treatment. Health related quality of life data for each participant are presented in Table 4. Participant 1, 4, and 5 reported high levels of physical, role, emotional, cognitive, and social function at baseline, which remained similar throughout DDAC treatment. Participant 2 reported an increase from baseline across all 5 functional domains during and 3-months post treatment. Participant 3 experienced a reduction and/or maintenance from baseline across all 5 functional domains at 4-weeks, with an increase from baseline across 4 of the 5 domains reported upon the completion of DDAC treatment. Participant 1, 4, and 5 reported minimal symptoms across the 9 symptom domains at all timepoints, with minor fatigue the most commonly reported. Participant 2 reported moderate fatigue, and moderate to high levels of dyspnea, insomnia, and constipation during and after DDAC treatment. Participant 3 experienced moderate to high symptoms across all symptom domains (excluding diarrhea), with symptoms across each domain predominantly worse at 4-weeks compared to 8-weeks (completion of DDAC). At all timepoints, perceived health and quality of life was slightly reduced in participant 1 and 4, moderately reduced in participants 2 and 5, and considerably reduced in participant 3.

**Table 4.** Health Related Quality of Life (QLQ-C30) Data at Baseline, 4-Weeks, 8-Weeks, and 20-Weeks for Each Participant.

	Functional scales					Symptom scales									Global
	Physical function, /100	Role function, /100	Emotional function, /100	Cognitive function, /100	Social function, /100	Fatigue, /100	Nausea, /100	Pain, /100	Dyspnea, /100	Insomnia, /100	Appetite loss, /100	Constipation, /100	Diarrhea, /100	Financial, /100	GHS/QoL, /100
Participant 1															
Baseline	86.67	83.33	83.33	100	100	11.11	0	0	0	0	0	0	0	0	83.33
4-weeks	93.33	100	83.33	100	100	11.11	0	33.33	0	33.33	0	0	0	0	58.33
8-weeks	80.00	100	66.67	100	100	33.33	0	0	0	33.33	0	0	0	0	83.33
20-weeks	93.33	100	100	100	83.33	11.11	0	0	33.33	0	0	0	0	0	83.33
Participant 2															
Baseline	86.67	33.34	50.00	50.00	66.67	55.55	0	100	66.67	33.33	0	66.67	0	33.33	25
4-weeks	100	66.67	83.33	66.67	83.33	66.67	16.67	33.33	66.67	33.33	33.33	66.67	0	0	58.33
8-weeks	93.33	66.67	91.67	66.67	66.67	66.67	0	33.33	66.67	66.67	0	100	0	0	50
20-weeks	86.67	66.67	83.33	83.33	83.33	66.67	0	33.33	66.67	66.67	0	33.33	0	0	58.33
Participant 3*															
Baseline	53.33	16.67	33.33	33.33	33.33	77.78	33.33	66.67	0	66.67	33.33	66.67	0	66.67	16.67
4-weeks	33.33	16.67	25.00	33.33	33.33	100	83.33	66.67	66.67	66.67	66.67	66.67	0	66.67	16.67
8-weeks	60.00	16.67	50.00	50.00	100	77.78	66.67	66.67	66.67	0	0	66.67	0	100	16.67
20-weeks	n/a	n/a	n/a	n/a	n/a	n/a	n/a	n/a	n/a	n/a	n/a	n/a	n/a	n/a	n/a
Participant 4															
Baseline	93.33	100	83.33	83.33	100	11.11	0	0	0	33.33	33.33	0	0	0	83.33
4-weeks	100	100	83.33	83.33	100	33.33	16.67	0	33.33	33.33	0	0	0	0	75
8-weeks	100	66.67	91.67	66.67	83.33	33.33	0	0	33.33	33.33	33.33	33.33	0	0	75
20-weeks	100	100	91.67	83.33	100	22.22	16.67	0	0	33.33	0	0	33.33	0	83.33
Participant 5															
Baseline	100	100	83.33	100	83.33	11.11	0	33.33	0	33.33	0	0	0	0	58.33
4-weeks	100	100	91.67	100	100	0	0	0	0	0	0	0	0	0	75
8-weeks	100	100	91.67	100	83.33	22.22	16.67	0	0	0	0	0	0	0	58.33
20-weeks	100	100	100	100	100	0	0	0	0	0	0	0	0	0	83.33

Abbreviations: QLQ-C30, quality of life questionnaire—cancer 30; GHS/QoL—global health score/quality of life; /100, score out of 100; n/a, participant did not attend.

* Participant 3 opted into the standard care arm and did not perform the exercise intervention.

## Discussion

This study investigated the impact of regular, individualized aerobic exercise on GLS, asymptomatic CTRCD incidence, aerobic fitness, and patient reported outcomes in a series of women undergoing DDAC chemotherapy for the treatment of breast cancer. Varied responses to exercise were observed for GLS, CTRCD incidence, and aerobic fitness. Exercise adherence was high for all participants, and no serious or non-serious adverse events or exacerbation of side-effects were experienced during exercise. Findings from this research outline the individual responses to exercise during DDAC treatment in women with breast cancer.

Of the 4 participants who completed the exercise intervention, 2 met the criteria for mild asymptomatic CTRCD, while 2 experienced an improvement in GLS upon the completion of DDAC. Participant 1, who experienced a 19% relative decline in GLS form baseline, was the eldest participant, and only participant with a cardiovascular risk factor for the development of CTRCD (hypertension), which may explain the decline in GLS observed.^13,33^ It is important to note that this decline occurred irrespective of participant 1 receiving the lowest cumulative AC dose (due to having the lowest body surface area), possibly indicating that lower cumulative AC doses may not always accommodate age related risks for CTRCD. Interestingly, GLS has been shown to be reduced in women with hypertension, however, participant 1 had the highest baseline GLS of all participants.^34,35^ Participant 4, who experienced a 20% relative decline in GLS from baseline, did not have any risk factors for the development of CTRCD, and was meeting the physical activity guidelines prior to commencing DDAC treatment. This was similar to participant 5, who in contrast, experienced an improvement in GLS from baseline upon the completion of DDAC. Both participants received a similar cumulative dose of DDAC and had similar adherence to exercise training frequency and intensity, but due to different immunohistology breast cancer characteristics, participant 4 received trastuzumab prior to DDAC. Along with cumulative AC dose, concomitant trastuzumab treatment with AC has been shown to increase the risk of developing CTRCD,^36,37^ which may explain this finding. Participant 5 also completed greater amounts of independent physical activity and reported less sedentary behavior during treatment, compared to participant 4, which may highlight the possible impact of adhering to an active lifestyle in conjunction with structured exercise on myocardial function during DDAC treatment.^38^ Participant 2, who was the youngest participant, experienced the greatest improvement in GLS. While risk factors for the development of CTRCD are well described, the findings observed highlight the individual nature of CTRCD.^13^ Along with reductions in GLS, participants 1 and 4 also had the greatest drop in LVEF, however, this drop was less than 10% for both. Although participant 3 (non-exercising participant) did not meet the criteria for mild asymptomatic CTRCD (14.3% decline in GLS from baseline), their GLS was significantly abnormal at baseline and at the completion of DDAC treatment, despite ceasing treatment after 3 cycles due to experiencing chemotherapy-related toxicity. It is unknown if GLS remained reduced 3-months post DDAC treatment as this participant did not return for their final visit. The reduction in GLS observed in participant 3 could be attributed to the relatively high AC dose across the 3 cycles combined with low levels of physical activity.

This study is the first to highlight the *individual* variation in GLS observed in response to AC treatment and aerobic exercise, although findings are similar to those presented at a group level by Foulkes et al^21^ and more recently Antunes et al.^39^ The same exercise intervention performed in this current study has been shown to improve GLS in women with cardiovascular disease risk factors,^22^ which may suggest that other factors, such cumulative AC dose and combination treatment outweigh the potential benefits of aerobic exercise on myocardial function as measured by GLS. Results from this study may also support this suggestion, as after 4-weeks, GLS was improved or maintained from baseline in all participants, potentially indicating an acute positive effect of exercise on GLS. However, at the completion of DDAC treatment when cumulative dose increased, GLS reduced below the 4-week measurement in all but one participant (Figure 1). These findings may indicate that 90 minutes of moderate intensity aerobic exercise per week can delay potential short term (<5 weeks) declines in GLS, but may not be sufficient to attenuate declines in GLS once cumulative AC dose peaks. This seems logical given cumulative AC is the single biggest risk factor for the development of CTRCD,^7,33,40^ although this finding must be interpreted with caution due to the nature of the case series design. Future research should explore whether higher volumes of moderate intensity aerobic exercise, or the inclusion of higher intensity interval training during DDAC treatment, can provide additional benefits beyond 4 weeks.

Similar to GLS, 2 exercising participants experienced a reduction in VO_2_peak, whilst 2 participants experienced a maintenance or improvement in VO_2_peak upon the completion of DDAC treatment. While participant 1 experienced a reduction in both GLS and VO_2_peak, participant 2, who experienced the greatest relative improvement in GLS from baseline, experienced the greatest reduction in VO_2_peak from baseline. Participant 5 was the only participant to experience an improvement in both GLS and VO_2_peak upon the completion of DDAC treatment. Although not an aim of this study, change in VO_2_peak and GLS did not appear to occur in the same direction, which is again similar to findings from recent randomized controlled trials.^21,39^ The reduction in VO_2_peak observed in 2 of the 4 exercising participants does not align with literature supporting the use of exercise to maintain or improve aerobic fitness in women with breast cancer undergoing AC chemotherapy.^20^ Participant 2, who experienced the greatest reduction in VO_2_peak upon the completion of DDAC, also scored poorer across multiple symptom domains in comparison to the other 3 exercising participants upon the completion of DDAC treatment (Table 4). As a VO_2_peak test is ceased at volitional exhaustion (ie, when the participant determines they have reached their maximum), an increase in symptoms could contribute to an increased perception of effort, resulting in a lower VO_2_peak. However, respiratory exchange ratio, RPE, and HR at 8-weeks were all higher than baseline testing in participant 2, suggesting a real reduction in VO_2_peak did occur, making this finding difficult to explain. The maintenance and improvement in VO_2_peak compared to baseline observed in participants 4 and 5 respectively, and better patient reported outcomes at baseline compared to participant 2, is a surprising finding given participant 4 and 5 received taxane-based and trastuzumab (participant 4 only) chemotherapy prior to commencing DDAC. It would be appropriate to assume the accumulation of treatment upon the completion of DDAC may have led to a reduction in VO_2_peak in participants 4 and 5, however, this was not observed. In comparison to age and gender-adjusted normative values for VO_2_peak, all participants were significantly below normative values at baseline and upon the completion of DDAC treatment.^41^ Participants 1 and 4, who met the criteria for mild asymptomatic CTRCD upon the completion of DDAC treatment, reached 53% and 65% of their age and gender predicted VO_2_peak at baseline, respectively. This may highlight reduced VO_2_peak at baseline as a potential risk for negative cardiovascular outcomes during DDAC treatment. This seems logical, given the strong relationship between VO_2_peak and heart failure, which is an end point of CTRCD.^42^ Furthermore, a VO_2_peak of <15.4 mL/kg/min has been shown to be associated with an increase in heart failure risk and mortality in breast cancer patients.^42,43^ Upon the completion of DDAC treatment, participant 1 had a VO_2_peak of 14.8 mL/kg/min, which may partially explain the decline in GLS observed. Participants 1 and 2 reported lower levels of exercise and physical activity in the 3 months following the completion of DDAC compared to participants 4 and 5, which is likely due to the continuation of taxane-based chemotherapy during this time, and may explain the changes in VO_2_peak observed at this timepoint.

The benefits of exercise and physical activity for improving fatigue, quality of life, and general treatment related experiences during chemotherapy are well recognized.^14,16,44^ In line with the literature, participant 3, who opted to not perform the exercise intervention during DDAC treatment, reported poorer outcomes within all domains of the functional, symptom, and global health scales of the QLQ-C30 questionnaire (Table 4), and within all domains of the QLQ-FA12 questionnaire (Table 3), compared to participants who completed the exercise intervention. Improved patient reported outcomes were also maintained 3-months post the completion of DDAC for all exercising participants, supporting the long-term benefits of exercising during chemotherapy. With no serious or non-serious adverse events of exacerbation of side-effects occurring during exercise, and the high adherence to exercise training frequency and intensity observed across all participants, findings from this study further support the safety and feasibility of exercise during DDAC chemotherapy for breast cancer. Despite the positive treatment related benefits experienced by exercising participants in this study, the low uptake of participants into the study (33.3% of those referred, enrolled in the study) is a key consideration for future research in this area. These findings suggest that exercise may not be valued or prioritized by women with breast cancer, there may be fear associated with exercising during breast cancer treatment, or there may be a lack of education about the benefits and safety of exercise during anti-cancer treatment provided by cancer clinicians. Such barriers to exercise participation have been identified in previous research,^45,46^ and highlight a potential lack in translation regarding the benefits of exercise to both consumers and clinicians.

### Strengths, Limitations, and Future Research

The small sample size prevented any formal statistical analysis being performed and impacts the generalization of findings to a larger population of women undergoing DDAC chemotherapy for the treatment of breast cancer. However, the study design allowed for detailed analysis at an individual level, with many of the key observations discussed likely to be missed if grouped analysis was performed. The varied participant characteristics at baseline, and different chemotherapy regimens prescribed adds additional benefits to the presentation of individual data, and more closely reflects clinical practice. Furthermore, with responses to both chemotherapy and exercise different for each individual, the presentation of individual data identified unique clinical considerations for exercise professionals prescribing exercise to this population, and for researchers planning future trials in this field. A true measure of VO_2_ maximum would have provided greater insight into the effect of exercise on cardiorespiratory fitness. However, this testing was not deemed appropriate in the population studied due to the requirement to do a validation stage, which adds additional participant burden. The supervised, one-to-one nature of exercise sessions performed in this study may not reflect the typical exercise performed during anti-cancer treatment that is, home-based exercise or group-based exercise where supervision is intermittent. As such, the exercise adherence achieved, and dose of exercise performed in this study may not be replicable in non-research settings. This study originally planned to recruit women with risk factors for the development of CTRCD, as literature in this area is lacking.^20^ However, due to the recruitment challenges described above, this was not achieved, and leaves a gap for future research to explore. While recent randomized controlled trials have investigated the impact of exercise on GLS and CTRCD incidence at a group level in this population,^21,39^ additional studies of similar design will assist in the validation of these findings. As the impact of exercise on GLS and asymptomatic CTRCD incidence was varied amongst participants in this study, future research should investigate if different volumes and/or intensities of exercise that is, up to 5 aerobic exercise sessions per week, or high intensity interval training, have a more consistent impact on GLS and asymptomatic CTRCD incidence. Future research may also monitor the effectiveness of group or home-based exercise on cardiac outcomes in women undergoing cardiotoxic chemotherapy for the treatment of breast cancer.

## Conclusion

This study provides insight into the impact of regular, individualized aerobic exercise on GLS, asymptomatic CTRCD incidence, aerobic fitness, and patient reported outcomes in a series of women undergoing DDAC chemotherapy for the treatment of breast cancer. Results highlight the individual variability in cardiovascular responses to exercise that can occur during DDAC treatment, and support the safety and efficacy of exercise during DDAC treatment. Findings can inform exercise professionals and researchers when designing individualized exercise programs for this population.
